# Evaluation of Ibuprofen Prolonged Release of Biomedical PLA-PEG-PLA Hydrogel via Degradation Mechanism

**DOI:** 10.1155/2023/5005316

**Published:** 2023-04-27

**Authors:** Hien Thi-Thanh Nguyen, Lam Thi-Truc Nguyen, Anh Cam Ha, Phu Dai Huynh

**Affiliations:** ^1^Vietnam National University Ho Chi Minh City, Linh Trung Ward, Thu Duc District, Vietnam; ^2^Faculty of Chemical Engineering, Ho Chi Minh City University of Technology, VNUHCM, 268 Ly Thuong Kiet Street, District 10, Ho Chi Minh City, Vietnam; ^3^Faculty of Chemical Engineering, Ho Chi Minh City University of Food Industry, 140 Le Trong Tan Street, Tan Phu District, Ho Chi Minh City, Vietnam; ^4^Center for German-Vietnamese Technology Academy, Ho Chi Minh City University of Food Industry, 140 Le Trong Tan Street, Tan Phu District, Ho Chi Minh City, Vietnam; ^5^Faculty of Materials Technology, Ho Chi Minh City University of Technology, VNUHCM, 268 Ly Thuong Kiet Street, District 10, Ho Chi Minh City, Vietnam; ^6^Polymer Research Center, Ho Chi Minh City University of Technology, VNUHCM, 268 Ly Thuong Kiet Street, District 10, Ho Chi Minh City, Vietnam

## Abstract

A micellar hydrogel has long been considered an intelligent hydrophobic drug delivery material. In this study, synthesized PLA_1750_-PEG_1750_-PLA_1750_ micellar hydrogel aims to encapsulate ibuprofen (IBU) in the core PLA hydrophobic of the micelle and prolong the drug release time by an injectable route. The structure and morphology of the PLA_1750_-PEG_1750_-PLA_1750_ copolymer hydrogel were demonstrated by ^1^H NMR and TEM data. The hydrogel also achieved a gel state at a high concentration of 25 wt.% under the physiological conditions of the body (37°C, pH 7.4). Besides, the biocompatibility test displayed that the hydrogel slightly affected mice after injection one week and fully recovered after four weeks. Furthermore, the *in vitro* degradation of the hydrogel showed apparent gel erosion after the first three weeks, which is related to the IBU release rate: slow for the first three weeks and then fast. As a result, the total drug release after three and four weeks was 18 wt.% and 41 wt.%, respectively. However, in the first 24 hours, the amount of the drug released was 10 wt.%, suggesting that the IBU drug diffused from the surface hydrogel to the buffer solution. These show that PLA_1750_-PEG_1750_-PLA_1750_ hydrogel can be a potential IBU drug delivery candidate.

## 1. Introduction

Ibuprofen (IBU) is a type of nonsteroidal anti-inflammatory drug. It has been commonly used for treating fever, pain, anti-inflammatory, and rheumatoid arthritis [1–5]. Treatment with ibuprofen can take several days. The oral administration route is a popular method of delivering the IBU drug because of its conveniences like safety, good patient compliance, and ease of ingestion. However, the poor aqueous solubility of IBU is a matter of concern. This leads to low dissolution and bioavailability. As known, IBU is a weak acidic drug with a pKa of 5.2 [1–3]. So, its solubility is limited in the stomach environment (pH 1–4). The crucial disadvantage causes difficulty controlling the desired concentration of the drug by oral delivery. Consequently, the most commonly used oral dose is 200–600 mg in 6 hours, whereas the required dosage for therapeutic effect in an adult is approximately 20–30 mg [1, 2, 4, 6]. That means the provided dose is more than many times the medicinal dose. Unfortunately, IBU may cause some adverse effects from a high oral dose, for example, gastrointestinal ulcers, congestive heart failure, or renal impairment [4–7]. Thus, finding a method to control the IBU content, especially in the case of prolonged therapy time, is an attractive topic.

Hydrogel is a three-dimensional hydrophilic polymer capable of absorbing a large amount of water or biological fluids. It is also known as an intelligent drug delivery system that can administrate drugs at the correct time, at the right site via an exact dose in the body. The main properties of the hydrogel are the ability to swell or shrink when stimulated by factors such as light, pH, temperature, and enzymes [8–10]. Significantly, hydrogel created from BAB amphiphilic copolymer is also a polymer micelle. The amphiphilic nature of the copolymer induces the polymeric chains' self-assembling to form a core/shell structure with a nanoscale average diameter [11–13]. As a result of this characteristic, this hydrogel can incorporate poorly water-soluble drugs. PLA-PEG-PLA amphiphilic BAB copolymer is a temperature-sensitive hydrogel with a micellar structure possessing a hydrophobic core (PLA) and hydrophilic shell (PEG). In addition, PEG and PLA approved by FDA (Food and Drug Administration) are polymers owning specific properties such as nontoxic, biocompatible, and biodegradable [12–15]. As known, the PLA-PEG-PLA temperature-sensitive hydrogel can apply as an injectable drug carrier, which can get a sol state at room temperature and a gel state of the physiological body conditions (37°C, pH 7.4) [11, 13]. So, PLA-PEG-PLA hydrogel is considered a potential candidate for IBU encapsulation in its network for orientation as an injectable delivery.

There are some main advantages in using this hydrogel as an injectable IBU delivery system. First, the solubility of IBU is increased because pH 7.4 of the hydrogel solution is very favorable for the dissolution of IBU, and the functional groups of IBU can also form hydrogen linking with PLA as a consequence of physical interaction [7, 12, 15]. These also make IBU keep on intact the drug molecules. Second, IBU hydrophobic drug encapsulated by the hydrogel PLA-PEG-PLA formulating as the injection can avoid the disadvantage of using soluble organic solvents, which can cause toxicity. The most important is that the IBU drug release rate can be controlled through the government of gel strength and degradation mechanism of the hydrogel. A good packing of a gel structure can indicate itself as a long-lasting sustained release profile because of resistance against gel erosion [16]. Note that the concentration, length of the PEG block and PLA block, and the ratio of PLA/PEG affect sharply on gel strength [11, 13, 16, 17]. In general, these mentioned parameters correlate reasonably with each other to create a firm gel under the physiological conditions of the body. Besides, the hydrophobic drug release mechanism of the PLA-PEG-PLA hydrogel mostly depends on the physical degradation/erosion of hydrogel caused by increasing water uptake of PEG to release some PEG moieties, the dissociation of PLA and diffusion drugs out of the porous structure [11–14, 18, 19]. However, the prolonged drug release by the decomposition mechanism prevails in the case of high hydrogel concentration, which causes increasing in crosslinking densities, and results in fewer pores, so the diffusion is reduced [20]. Therefore, PLA-PEG-PLA hydrogel with high concentration can increase the chances of extending the release of the IBU drug and controlling its content. All of those suggest that the IBU formulation for the injection route by PLA-PEG-PLA hydrogel delivery system may not need significant excess of the drug concentration compared to the therapeutic dose, safety, and may prolong the release time.

The main goal of this journal is to synthesize PLA-PEG-PLA copolymer obtained firm gel at 37°C and pH 7.4 at high concentration (20 wt.%–30 wt.%). The degradation of PLA-PEG-PLA copolymer *in vitro* and *in vivo* (in both cases, present or absence, of IBU) was analyzed carefully to understand the degradation characteristic thoroughly. From there, the correlation between the rule of degradation and drug release rate was clarified via the *in vitro* IBU release experiments. Besides, the chemical structure, composition, and morphology, size of the copolymers were demonstrated by ^1^H NMR spectra and TEM, respectively. Sol-gel behavior of the hydrogel was examined by a tube inverting method. The property of biomedical materials like biocompatibility was evaluated by *in vivo* testing.

## 2. Materials and Methods

### 2.1. Materials and Animals

Polyethylene glycol (PEG) (Mn = 1750 g·mol^−1^), stannous octoate (Sn (Oct)_2_), and phosphate-buffered saline (PBS) were purchased from Sigma-Aldrich. D, L-lactide was bought from Tokyo Chemical Industry. Ibuprofen and adult male Swiss Albino mice (*Mus musculus*) (25–30 g) were obtained from the Institute of Drugs Quality Control in Ho Chi Minh City (Vietnam).

### 2.2. Methods

#### 2.2.1. Synthesis of PLA-PEG-PLA Triblock Copolymer

PLA-PEG-PLA triblock copolymer was synthesized by ring-opening of D, L-lactide in the presence of PEG and using stannous octoate as a catalyst, following a procedure described previously [13, 14, 21]. Firstly, PEG (5 g) and stannous octoate (0.065 g) were introduced into a flask and heated in a vacuum at 110°C for 2 hours to eliminate the trace amount of water. After that, the flask was cooled to 60°C to add the D, L-lactide (13 g). The polymerization occurred in a nitrogen gas environment at 130°C for 20 hours. Finally, the copolymer was collected by precipitate in excess diethyl ether and dried under vacuum at 45°C for 48 hours.

#### 2.2.2. Characterization

*(1)* ^*1*^ *H-NMR (ProtonNuclear Magnetic Resonance) Analysis.* ^1^H-NMR was applied for compositional and structural analyses of the freeze-dried triblock copolymer. The spectra were recorded from the Bruker Advance machine at 500 MHz with D_2_O or CDCl_3_, adding 0.03% (v/v) tetramethylsilane (TMS) as a solvent signal.

*(2) TEM (Transmission Electron Microscope) Analysis.* The morphology of the copolymer observed by the TEM image was performed on JEM 2100, Jeol, Japan. The copolymer solution was put on a copper grid and dried at room temperature before measurements.

#### 2.2.3. Sol-Gel Phase Transition Measurement

Sol-gel transition behavior was conducted via the test tube inverting method. The sol state was characterized by a liquid state, opposite to the gel state. In the beginning, the copolymers were solubilized in PBS buffer (pH 7.4) at the specified concentration in 4 mL vials and stored at 10°C for 12 hours. Then, the vials were placed in a water bath in which the temperature was set to moderately increased temperature from 15°C to 60°C with a step of 1°C/min and kept at each temperature for 5 min. The sol-gel phase transition was observed visually by inverting the vials. The gel state was defined without flow in 10 s [13, 21].

#### 2.2.4. *In Vitro* Degradation of Copolymers

The polymeric aqueous solutions (25 wt.% in PBS and initial pH 7.4) were incubated at 37°C for 1 hour to form gels in tubes. After that, 3 mL of the buffer solution (pH 7.4) was put on top of the gels. Each week, the gels were observed, photographed, and the aqueous solutions measured pH by a pH meter [21].

#### 2.2.5. *In Vivo* Degradation and Biocompatibility of Copolymers

The experiments were performed via dorsal subcutaneous copolymer administration in mice. 0.1 mL of the triblock copolymer with 25 wt.% concentration in aqueous solution (PBS solution and pH 7.4) was quickly injected into the mice by a syringe with a 23-gauge needle at room temperature. At a predetermined time, the mice were sacrificed, and the formed gels on their body were photographed and collected. Furthermore, the histocompatibility of the copolymers was determined by a histological study. The tissue surrounding the subcutaneous injection sites was surgically taken off to test hematoxylin-eosin staining (HE) for histopathological examination. All *in vivo* testing was executed by the guidelines of the Vietnam National University for animal experiments.

#### 2.2.6. *In Vitro* IBU Release

The copolymer solution (25 wt.% and pH 7.4) and IBU (2 wt.% based on the weight of the copolymer) were dissolved in a vial (5 mL) and stirred for 12 hours at 10°C. After, the mixture was equilibrated at 10°C for 12 hours and incubated at 37°C for 1 hour to create the gel. Then, the vial was added 3 mL of PBS (pH 7.4). At given time points, 1.5 mL of the solution was removed for the analysis, and 1.5 mL of fresh PBS was replaced in the releasing tube. The IBU content was measured by high-performance liquid chromatography (HPLC) with the C18 column (5 *μ*m, 4.6 mm × 250 mm) and the UV detector at 223 nm absorbance [22].

## 3. Results and Discussion

### 3.1. Characterization of PLA-PEG-PLA Copolymer

**Figure 1:**
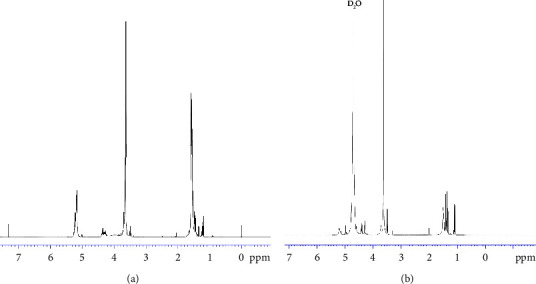
^1^H NMR spectra of PLA-PEG-PLA copolymer: (a) in CDCl_3_ and (b) in D_2_O.

The synthesized PLA-PEG-PLA copolymer was analyzed structure by the ^1^H-NMR. As shown in Figure 1(a), the chemical shift *δ* (ppm) of the ^1^H-NMR spectrum in CDCl_3_ presented at 5.2 ppm (1H, CO-CH (CH_3_)-O), 1.5 ppm (3H, CO-CH(CH_3_)-O) of PLA, and 3.6 ppm (4H,-CH_2_-CH_2_-O-) of PEG. Note that the polymerization degree (DP) of each PLA block in the structure and the molar weight of the triblock copolymer were calculated by comparing the intensity of the LA (lactate units) characteristic resonance at 5.2 ppm and that of EO (ethylene oxide units) at 3.6 ppm in the NMR data [14]. As a result, the PLA_1750_-PEG_1750_-PLA_1750_ was obtained. In Figure 1(b), ^1^H-NMR spectroscopy of PLA-PEG-PLA copolymer in D_2_O solvent displayed the protons of PEG (-CH_2_-CH_2_-O-) with intensity the same as in the CDCl_3_ solvent. By contrast, protons of methyl and methine signals of PLA in the D_2_O solvent were lower than in the CDCl_3_ solvent. D_2_O is not a good solvent for the PLA block by its hydrophobic characteristic. As known, in selective solvents, only the representative signals of the soluble block can be noticed, whereas those of the insoluble block are difficult to detect [13, 23, 24]. It suggested the PLA situated in the core of the micellar PLA-PEG-PLA structure was caused by decreasing its interaction with the solvent. Meanwhile, the PEG was located externally in a mobile state.

**Figure 2:**
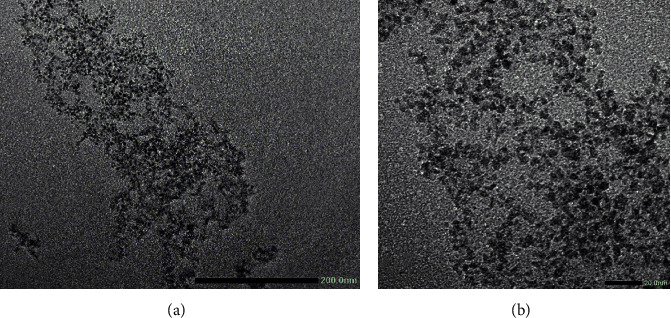
TEM images of PLA_1750_-PEG_1750_-PLA_1750_ copolymer: (a) high magnification and (b) low magnification.

In addition, the morphology of the PLA_1750_-PEG_1750_-PLA_1750_ copolymer is shown in Figure 2 as a spherical shape. The average size of the triblock copolymer was under 10 nm in diameter. All of these demonstrated that the formed PLA_1750_-PEG_1750_-PLA_1750_ copolymer is a type of micelle.

### 3.2. Sol-Gel Phase Transition

**Figure 3:**
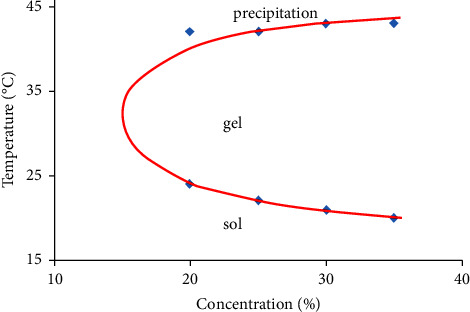
Sol-gel phase transition diagram of PLA_1750_-PEG_1750_-PLA_1750_ copolymer.

The PLA_1750_-PEG_1750_-PLA_1750_ copolymer was dissolved in PBS at a pH of 7.4 with different concentrations to form the aqueous solutions. It was found that the copolymer had the sol-gel transition phase depending on various concentrations and temperatures. The sol-gel diagram is *U* shaped in Figure 3. It displayed the curve of the lower critical gelation temperature (LCGT) points and the upper critical gelation temperature (UCGT) points. In observation, there are three parts in the diagram, including the sol phase under the LCGT curve, the gel phase between the LCGT and the UCGT curves, and the precipitation phase above the LCGT curve. With increasing concentration, the LCGT values of the copolymers decreased, which is appropriate to the trend of the previous report [21]. However, the UCGT points of the copolymer do not significantly change (about 42°C). The results can explain that the gelation process is governed by interchain hydrophobic interactions of PLA segments of the copolymer or critical gel concentration (CGC) of the copolymer. With a high proportion of the PLA hydrophobic, the self-assembly of PLA_1750_-PEG_1750_-PLA_1750_ amphiphilic molecules to form gel is taken either at a given temperature by increasing the concentrations beyond the CGC or at a given concentration by increasing the temperatures over the LCGT values. But, heating over the UCGT point, gels are dehydrated, and the copolymer transfers to the precipitation phase. Attentively, it was a phenomenon of extruding water out of the copolymer and reducing gel size.

The chart of sol-gel behavior also shows the high content of PLA_1750_-PEG_1750_-PLA_1750_ copolymer from 20 wt.% to 35 wt.% concentration capable of gelling at 37°C. Therefore, the PLA-PEG-PLA copolymer is a hydrogel. In those concentrations, the hydrogel of 25 wt.% was selected for further study because the gel was more stable in a wide temperature range than the concentration of 20 wt.%. The hydrogels of 30 wt.% and 35 wt.% concentrations were neglected due to disadvantages such as needle blockage during injection and long-time decomposition.

### 3.3. *In Vitro* Degradation of the PLA_1750_-PEG_1750_-PLA_1750_ Hydrogel

**Figure 4:**
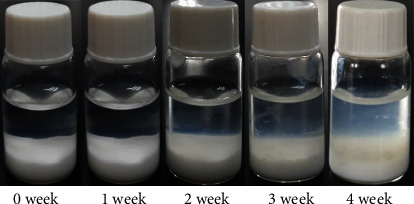
Images of PLA-PEG-PLA gels in the *in vitro* degradation experiments.

The *in vitro* degradation experiments of the PLA_1750_-PEG_1750_-PLA_1750_ hydrogel (25 wt.%) were recorded by observing the gel state of the hydrogel in the buffer solution (initial pH 7.4) at 37°C.  Figure 4 shows the images of the gels photographed from the initial week to the fourth week. For two weeks, the gels were retained as shown in the original white color and did not change in volume. The aqueous solution became opaque in the third week, but the gel did not change its volume and shape. After four weeks, the gel surface was eroded, which led to the cloudy buffer solution. This phenomenon was due to the PEG water uptake and release as the mode of surface erosion and PLA degradation as bulk erosion [18, 19]. Remarkably, the pH value of the solution also decreased during the degradation time in Table 1. The pH change of the buffer medium dropped quickly from 7.40 to 3.66 in the first week, while it reduced slightly in the second week that resulted from the generation of lactic acid as a degradation product of the hydrogel.

**Figure 5:**
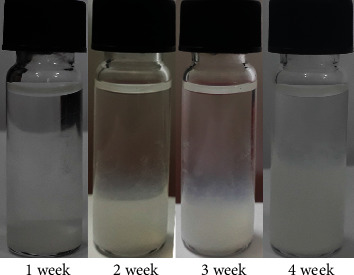
Images of IBU-loaded gels in the *in vitro* degradation experiments.

However, in the presence of IBU in the hydrogel, the white color of the solution changes, not transparent after two weeks, as shown in Figure 5. The formed cloudy liquid on the gel showed evidently after three weeks. It suggested that the drug on the surface of the gel combined with the chain cleavage in the amphiphilic polymers that diffused together into the solution, especially the insoluble feature of IBU in low pH solution, or the decrease of gel strength by the intrusion of IBU into the structure of the gel.

### 3.4. *In Vivo* Degradation and Biocompatibility of the Hydrogel

**Figure 6:**
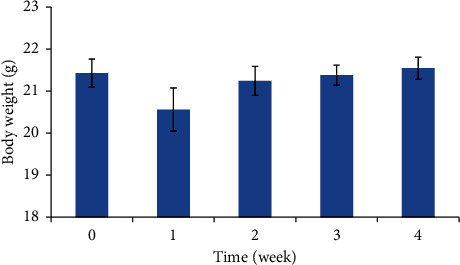
Weight of mice after injecting the hydrogel at different times.

Mice were injected subcutaneously with 100 *μ*L of the PLA_1750_-PEG_1750_-PLA_1750_ hydrogel solution (25 wt.%). They were weighted weekly in the results, as shown in Figure 6. Realistically, the mice lost weight lightly in the first week, about 1 g per mouse. Their weight increased slightly in the second week and remained stable for the next weeks. It can be explained that the hydrogel affected a little the immune system of the mice as the response of their body to a foreign substance. However, in observation, the mice still normally acted and did not change their appearance for four weeks.

**Figure 7:**
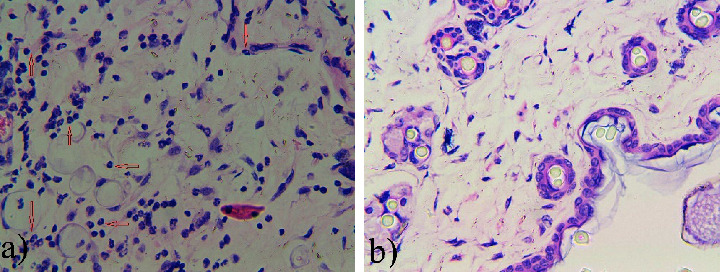
Microscopic photos of surrounding tissues at the dorsal subcutaneous sites experimented with HE-staining: (a) 1 week and (b) 4 weeks.

In addition, HE-staining experiments of surrounding tissues at the sites dorsal subcutaneous after implanting the PLA_1750_-PEG_1750_-PLA_1750_ solution (in PBS and pH 7.4) into mice were performed for the inflammatory response. As results in Figure 7, in the first week, the tissue description displayed an infiltrate occupied by neutrophils and lymphocytes as characteristic of acute inflammation. Importantly, the concentration of neutrophils and lymphocytes was not enough to evaluate the severity of the tissue. At four weeks, the number of neutrophils and lymphocytes reduced sharply.

Generally, the hydrogel slightly impacted mice in the initial time as a response of the immune system to attack a stranger substance infiltrated into a body. However, the mice recovered weight, as well as inflammatory tissues. Therefore, it recommends that the hydrogel is compatible with the mice.

**Figure 8:**
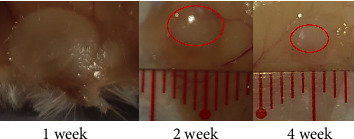
Images of the gels in the *in vivo* degradation experiments.

The *in vivo* degradation of the PLA_1750_-PEG_1750_-PLA_1750_ hydrogel displayed in Figure 8 indicates the decrease in volume and size of the gel over time. In observation, the formed gel was a large, rigid piece after one week. It exhibited the stability of the hydrogel. After two weeks, the amount of gel was obviously narrowed, and the remaining gel after four weeks was little bit. This mass change seems to follow the rule of degradation of the gel *in vitro* testing that it reduced sharply the volume after four weeks.

**Figure 9:**
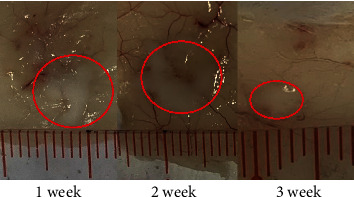
Images of IBU-loaded gels in the *in vivo* degradation experiments.

However, the situation of the gels containing IBU in Figure 9 is slightly different from those without the drug in Figure 8. These gels exhibited low mechanical strength after two weeks. Besides, the gel volume and mass decreased quickly after three weeks and almost disappeared after four weeks. It can be explained by the gel's structural integrity and mechanical properties that can be affected by the change of concentration hydrogel due to the presence of the drug and the incorrect physical cross-linking between IBU and PLA. This result correlates with the *in vitro* degradation assay in Figure 5 that gel damage appeared after two weeks.

Based on the *in vivo* and *in vitro* degradation results, PLA_1750_-PEG_1750_-PLA_1750_ gels corrosion initially occurred by degrading hydrophilic PEG blocks and hydrolysis of acid lactide. As a result, the pH of the solution decreased, and the gel narrowed and deteriorated. In the *in vitro* experiments, the visual gel observation did not detect a difference because only degraded small fractions of PEG were diffused and were well soluble in the solution after the second week. Meanwhile, cleaved segments of PLA could be trapped in the network and were continued decomposition. After that, formed smaller PLA chains could diffuse from the bulk gel to make the cloudy liquid. In Table 2, the LA/EO ratio of the lyophilized hydrogel after two weeks of *in vitro* degradation was higher than that of the initial hydrogel. This indicated more loss of PEG from the hydrogel in the initial time. It is an advantage for IBU release control due to linking IBU to PLA placed in the core of the hydrogel, except IBU on the surface of the gel. However, the rate of *in vivo* degradation, especially in the case of IBU presence, was faster than that of *in vitro* degradation because of enzymatic degradation, cytophagocytosis apart from the hydrolysis, and poor gel strength [25]. It is worth considering further *in vivo* degradation because of the effect on the bioavailability of the drug.

In brief, a prediction is that the *in vitro* drug release rate can occur slightly in the first three weeks and strongly in the next weeks. Regarding the IBU on the surface of the gel, the release process may occur as a burst drug phenomenon at the initial time. However, if the IBU is encapsulated and held perfectly in the core of the high-concentration hydrogel (25 wt.%), the drug release will be controlled.

### 3.5. *In Vitro* IBU Release

**Figure 10:**
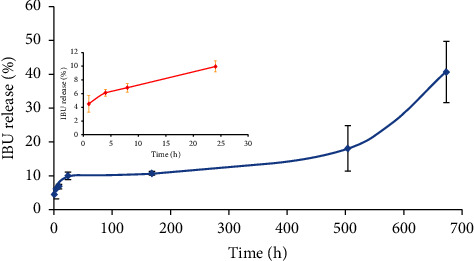
*In vitro* IBU-release profile of PLA_1750_-PEG_1750_-PLA_1750_ hydrogel in PBS (pH 7.4).

As illustrated in Figure 10, the profile reveals IBU drug release for 672 hours (4 weeks). The drug release process can be divided into three stages. In the initial 24 hours, the drug release rate increased quickly with 10 wt.% concentration let into the buffer solution as the result of the drug at the surface of hydrogel, without encapsulation in the core of micelles. In the second stage, the release rate occurred slowly for the next 504 hours (3 weeks) in the amount of drug release at 18 wt.% (adding 8 wt.% after 24 hours). Finally, the release rate increased fast from 504 hours to 672 hours (4 weeks) with the IBU accumulation release of 41 wt.% (increasing 23 wt.% after 504 hours). It is consistent with the prediction *in vitro* degradation analysis of the copolymer. It can be said that the initial 24-hour IBU release stage was the diffusion process. After that, the drug release process was controlled by the degradation of the hydrogel. In the initial three weeks, the hydrogel degradation process was not fast, so the content of the drug was released small. After three weeks, the hydrogel erosion process was intense, which caused the drug release rate to increase rapidly. Importantly, all the stages of the release process did not show a burst release phenomenon. Compared with the commercially available IBU capsule on the commercial market Fenlong-SR 400 mg, which can release 100% of the drug for about 210 minutes in the *in vitro* release experiments, PLA_1750_-PEG_1750_-PLA_1750_ hydrogel displayed more sustained release characteristics [26]. Note also that the *in vivo* degradation rate in the presence of IBU was faster than that of the *in vitro*. Therefore, the *in vivo* drug release results can be different from the *in vitro* test. However, the rapid deterioration of IBU-loaded gel occurred from the second week, so the hydrogel still can become the potential IBU prolonging delivery system.

## 4. Conclusions

The PLA_1750_-PEG_1750_-PLA_1750_ copolymer hydrogel as a micelle used to release the IBU hydrophobic drug has denoted a promising result. The copolymer achieved the obligatory requirements of the injectable hydrogel carriers. It performed the firm gel state at 37°C and pH 7.4. The biocompatibility characteristic of the hydrogel was displayed by the *in vivo* experiments in which the inflammatory tissues at the injected site in the body mice recovered evidently after four weeks. The degradation process of the copolymer occurred slowly in the initial three weeks and rapidly in the fourth week as shown in the *in vitro* tests. The slow deterioration combined with the holding of the IBU drug inside the PLA hydrophobic core of the micellar polymer led to the slow drug release. After four weeks of *in vitro* drug release, the amount of the drug was released at 41 wt.%. In there, 10 wt.% drugs were diffused from the surface of the hydrogel in the initial 24 hours. Concerning the *in vivo* degradation results, the gel erosion was faster than that of *in vitro* related to gel strength. It is necessary to study the interaction of the drug in the hydrogel structure that influences the gel strength. In general, these obtained results prove that PLA_1750_-PEG_1750_-PLA_1750_ hydrogel can prolong IBU release.

## Figures and Tables

**Table 1:** Change in pH value of the copolymer solution in time.

	Initial time	1 week	2 weeks	3 weeks	4 weeks
pH	7.40	3.66 ± 0.06	3.34 ± 0.04	3.24 ± 0.04	3.20 ± 0.03

**Table 2:** Variations of LA/EO ratio of PLA_1750_-PEG_1750_-PLA_1750_ in the *in vitro* degradation time.

Degradation time (week)	LA/EO^a^
0	1.21
2	1.27

(^a^Calculated from the integrations of ^1^H NMR bands belonging to PEG blocks at 3.6 ppm and to PLA blocks at 5.2 ppm).

## Data Availability

The data used to support the findings of this study are included within the manuscript.
